# Sensory Deprivation and the Brain: Neurobiological Mechanisms, Psychological Effects, and Clinical Implications

**DOI:** 10.3390/brainsci16020122

**Published:** 2026-01-23

**Authors:** Donatella Marazziti, Gerardo Russomanno, Matteo Gambini, Francesca Rita Digiuseppe, Enrico Fazio, Riccardo Gurrieri

**Affiliations:** Department of Clinical and Experimental Medicine, Section of Psychiatry, University of Pisa, 56100 Pisa, Italygambinimatteo1996@gmail.com (M.G.); dgsfrn@gmail.com (F.R.D.); riccardogurrieri4@gmail.com (R.G.)

**Keywords:** sensory deprivation, neuroplasticity, neuroinflammation, circadian rhythms, extreme environments, spaceflight, psychopathology, mental wellbeing

## Abstract

**Background/Objectives:** Sensory deprivation, defined as a reduction or absence of external sensory input across one or more modalities, has long been investigated in extreme and experimental settings. More recently, its relevance has expanded to clinical contexts and environmental conditions. The present narrative review aims to synthesize current evidence on the neurobiological mechanisms, psychological effects, and clinical implications of sensory deprivation, with particular attention to its dual role as both a risk factor and, under controlled conditions, a potential therapeutic tool. **Methods:** A narrative literature search was conducted using PubMed, Scopus, and PsycINFO, covering studies published up to August 2025. Search terms included sensory deprivation, neuroplasticity, neurotransmitters, HPA axis, neuro-inflammation, circadian rhythms, psychopathology, extreme environments, and spaceflight. Preclinical and clinical studies examining biological, cognitive, and psychological consequences of reduced sensory stimulation were included. Data were synthesized thematically without quantitative meta-analysis. **Results:** Evidence indicates that sensory deprivation induces widespread neurobiological adaptations involving neurotransmitter systems (particularly dopaminergic pathways), dysregulation of the hypothalamic–pituitary–adrenal axis, neuroimmune activation, circadian rhythm disruption, and structural and functional brain changes, notably affecting the hippocampus. These alterations are associated with increased vulnerability to depression, anxiety, hallucinations, dissociative symptoms, and cognitive impairment. Duration, voluntariness, and individual differences (e.g., baseline vulnerability/resilience, trait anxiety, and prior psychiatric history) critically modulate outcomes. However, short-term and voluntary sensory restriction, such as Floatation-REST, may promote relaxation and emotional regulation under specific conditions. **Conclusions:** Sensory deprivation exerts complex, context-dependent effects on brain function and mental health. Duration, individual vulnerability, and voluntariness critically modulate outcomes. Understanding these mechanisms is increasingly relevant for clinical practice and for developing preventive strategies in extreme environments, including future long-duration space missions.

## 1. Introduction

Sensory deprivation refers to a reduction or absence of external sensory input across one or more modalities (visual, auditory, tactile/kinesthetic, olfactory, and proprioceptive), which may exert profound effects on human brain and behavior. Traditionally studied in extreme conditions such as solitary confinement or sensory isolation chambers [1,2], sensory deprivation has gained renewed scientific interest due to its broader relevance across clinical (e.g., intensive care units, ICU), environmental (e.g., spaceflights, polar expeditions, submarines, sea surviving in a raft, cave explorations), and experimental (e.g., Floatation-REST) contexts [3,4,5]. Despite its heterogeneity, converging evidence suggests that altered sensory exposure might significantly modulate key biological systems involved in emotional regulation, cognitive functioning and mental wellbeing [6,7,8].

From the neurobiological perspective, sensory deprivation is supposed to trigger large-scale neural adaptations involving both functional and structural plasticity. Animal and human studies demonstrated that a lack of sensory input in one modality might lead to cross-modal recruitment of cortical areas and reorganization of synaptic connectivity [9,10]. However, plasticity is not always adaptive, as sensory deprivation can disrupt excitatory–inhibitory balance in cortical and subcortical circuits, destabilizing neurotransmission and altering neurochemical signaling, in particular that of dopamine circuits [11,12]. The resulting cascade dysfunctions have been associated with increased vulnerability to depression and emotional instability, particularly when combined with social isolation [13]. Prolonged sensory or social isolation in rodents has been shown to activate microglia, the brain’s resident immune cells, especially in the prefrontal cortex, hippocampus, and nucleus accumbens [14]. Elevated expression of pro-inflammatory cytokines (e.g., tumor necrosis factor-α: TNF-α; interleukin1β: IL-1β) with concurrent changes in astrocyte phenotype paralleling the microglial response, while suggesting a chronic neuro-inflammatory state [15]. Similar patterns of inflammation-related gene expression have been identified in the human brain [16,17].

All together, these neurobiological alterations are hypothesized to underlie many of the affective, cognitive and psychopathologic symptoms observed during or after sensory deprivation, in particular, anxiety, derealization, mood lability, depression, and even hallucinatory or transient psychotic-like experiences after prolonged or multisensory deprivation [18,19]. These outcomes are common in ICU patients, astronauts, prisoners, and other individuals exposed to environments of extreme sensory restriction. However, their occurrence and severity may vary according to the individual basis and if the sensory deprivation is voluntary or not [3,20,21].

Interestingly, controlled and time-limited sensory deprivation, typically lasting from 30 min to several hours per session, may promote psychophysiological restoration. Floatation-REST, which isolates individuals in dark, soundproof saltwater tanks, has been associated with reduced anxiety, enhanced interoception, and improvements in perceived mental wellbeing [22,23]. These effects appear to be mediated by reductions in sympathetic arousal and lower cortisol levels, suggesting that under specific conditions, sensory restriction may foster relaxation and emotional regulation. Interestingly, in ancient cultures, sensory deprivation was often used for spiritual or therapeutic purposes. It is noteworthy that in the ancient Greece, isolation was one of the treatments of insanity; in Rome, Celsus recommended dark room confinement for mental health, with varying results [24]. Similarly, Tibetan Buddhism uses “dark retreats” for deep introspection [25].

The duality of sensory deprivation as both a potential risk factor and a therapeutic strategy raises important questions about its mechanisms and boundary conditions. Given the current ongoing projects of distant space missions and explorations of exceptional environments that should ensure the maintenance of adequate sensory stimulations, it seems worthwhile and timely to deepen the effect of sensory deprivation. Therefore, the present narrative review aims to critically synthesize existing studies on the neurobiological effects of sensory deprivation and its impact on psychological wellbeing and psychopathology. Particular attention will be given to the available literature on absent sensory stimulation across both preclinical and clinical models. Further, the review will discuss emerging insights into sensory deprivation experienced amongst people in extreme environments and astronauts.

## 2. Methods

Given the conceptual and methodological heterogeneity of the literature (ranging from unimodal sensory loss to multisensory restriction and ICE/spaceflight analogs with multifactorial exposures), we conducted a narrative review aimed at mapping converging mechanisms and clinically relevant patterns. The search strategy involved the use of electronic databases, including PubMed, Scopus, and PsycINFO. Search strings were developed using Boolean operators (AND/OR) combining the following keywords across databases: “*Sensory Deprivation*”, “*Biological Effects*”, “*Neurobiology*”, “*Neuroplasticity*”, “*Neurotransmitters*”, “*HPA axis*”, “*Neuroinflammation*”, “*Wellbeing*”, “*Psychopathology*”, “*Depression*”, “*Hallucinations*”, “*Social Isolation*”, “*Extreme environmental conditions*”, “*Imprisonment*”; “*Interrogation/Torture*”; “*Spaceflight*” and “*Astronauts*”. There were no restrictions on publication year, and the literature was considered up to August 2025.

Both original experimental/clinical studies and relevant narrative or systematic reviews were considered eligible when they provided data on biological, psychological, or clinical consequences of reduced sensory stimulation. Studies were included if they investigated the biological, psychological, or cognitive consequences of reduced or absent sensory stimulation, either in naturalistic (e.g., space missions, ICU) or experimental (e.g., sensory isolation chambers) contexts. Exclusion criteria were articles not in English, conference abstracts without full data, and studies not primarily focused on sensory deprivation. Given the narrative nature of the review, articles were screened in multiple stages to ensure thematic relevance. Titles and abstracts were first examined to identify studies addressing reduced sensory input or sensory monotony in biological, psychological, or clinical contexts. Full texts were then evaluated for conceptual coherence with the scope of the review. Studies were grouped thematically (neurobiology, circadian rhythms, immune effects, structural brain changes, psychopathology, and extreme environments). No formal quantitative assessment or risk-of-bias scoring was performed, consistent with the narrative design.

Particular attention was paid to converging neurobiological mechanisms across models, and to psychological outcomes with translational relevance. Extreme environments were defined as isolated, confined, and operational contexts characterized by reduced sensory variability, environmental monotony, limited social interaction, and altered external zeitgebers. These settings included spaceflight and space analogs (e.g., MARS-500), Antarctic stations, submarines, cave expeditions, and comparable environments where prolonged exposure leads to sensory under-stimulation rather than complete sensory absence. No strict minimum duration was imposed, as the literature encompasses both acute experimental paradigms (minutes to hours; e.g., Floatation-REST, anechoic chambers) and prolonged naturalistic conditions (days to months; e.g., confinement, polar expeditions, space missions). Effects are therefore discussed according to duration and context rather than predefined temporal thresholds. Given the broad and interdisciplinary nature of the topic, this review does not aim to provide a systematic quantification of effects, but rather to outline key conceptual domains and identify gaps for future research.

## 3. Biological Effects of Sensory Deprivation

### 3.1. Neurotransmitters

A recent study in a mouse model showed that even 24 h of sensory deprivation (e.g., closing the nostrils to eliminate olfactory input) causes rapid changes in the expression of key enzymes in the synthesis of dopamine, namely dopa-decarboxylase (DDC) and tyrosine hydroxylase (TH), in the olfactory cortex [26]. After a transient decrease in DDC activity, the next day, a significant reduction in TH levels was detected that became more relevant after three days, probably to adapt dopaminergic production to rebalance neuronal activity [26]. Another study showed that sensory experience, and its absence, directly affects dopaminergic gene transcription in the somatosensory cortex (barrel cortex) [27]. Interestingly, the tactile input was found to “compete” with deprivation to regulate the expression of dopamine-related genes [27].

Direct data on the effect of total sensory deprivation on dopaminergic activity in the human brain is still limited. A study with positron emission tomography (PET) with [(11)C]-raclopride, a tracer binding to dopamine D2/D3 receptors, showed that sensory stimulation did not significantly change dopamine receptor availability, suggesting that this system may be relatively stable under normal stimulus conditions. However, no PET or fMRI studies are available that have directly measured dopaminergic activity during conditions of prolonged sensory deprivation (such as isolation in anechoic chambers or flotation tanks in total absence of stimuli). This is partly due to the ethical and practical difficulties in carrying out neuroimaging studies amongst subjects living in extreme conditions.

Some indirect research, such as that on prolonged social isolation (which can be seen as a partial form of sensory deprivation), showed alterations in dopaminergic reward circuits, similar to those observed in conditions of stress or depression [28,29,30,31]. Isolation may reduce the dopaminergic response to rewarding stimuli, as shown in a study on 40 healthy adults exposed to 10 h of social isolation and 10 h of fasting [12]. At the end, the researchers observed a selective activation of reward-related midbrain areas, the VTA, in response to social stimuli, similar to the brain’s response to images of food after fasting. Social isolation, thus, would generate a kind of “social craving”. Finally, neuroimaging studies on chronic loneliness show a decreased activation of the ventral striatum, the main dopaminergic region involved in reward, accompanied by high levels of cortisol, which has a depressing effect on dopaminergic activity [12]. Although direct evidence in human beings remains partial, the results converge to suggest that the brain perceives social isolation as motivational deprivation, while activating (or depressing) dopaminergic circuits linked to reward and stress. Although social isolation does not represent total sensory deprivation, it markedly reduces socially driven sensory input and environmental complexity and is therefore discussed here as an adjacent model.

To summarize, dopamine may act as a key modulatory component in the brain’s adaptive recalibration to environments with reduced stimulation, interacting with other neuromodulatory systems to influence motivation, salience attribution, and learning processes.

### 3.2. The HPA Axis

Rodent studies indicate prolonged social isolation may provoke increased corticosterone levels and hyperactivation of the HPA axis [32,33,34,35,36,37,38].

In humans, if short sessions of sensory deprivation do not seem to impair the HPA axis [39,40], longer periods may induce negative effects. Seminal studies conducted on people kept in confinement for long periods show mixed data on cortisol and ACTH levels and their circadian patterns [41,42,43,44,45]. Data from the MARS 500 Project indicate that although at 105 days there was no significant increase in HPA axis activation, data were quite different at 520 days [46,47,48]. Indeed, the volunteers undergoing a 520-day confinement showed a significant increase in salivary cortisol during the isolation period associated with a reduction in cortical activity detectable on the EEG. However, moderate physical exercise produced possible compensatory effects [49].

Other data on the activation of HPA under sensory deprivation conditions have been gathered from research conducted on speleologists and Antarctic expeditioners [50]. Before cave entry, there is a marked elevation in serum cortisol even in elite cavers, indicating an anticipatory stress response. This initial spike in cortisol is reportedly reduced over time as the cavers acclimate to the environment. Notably, prolonged cave exploration also triggers an increase in growth hormone (GH) production, suggesting a compensatory hormonal response. After the exit from the cave, cortisol levels decrease, and GH levels are restored within 24 h, reflecting a recovery from the environmental and physiological stressors of the expedition [51,52,53]. During Antarctic expeditions, several studies observed an increase in average cortisol levels returning to baseline levels at the end of the mission [50,54,55,56,57], with stress-reducing practices such as yoga that demonstrated and promoted this adaptation [57,58]. Again, the influence of altitude was noted in cortisol, endocannabinoids and catecholamine patterns with differences between crews staying at Neumeyer III (sea level) or Concordia station located at 3232 m [59]. Increased levels of norepinephrine and thyroid hormones were reported and related to cold adaptation [50].

Taken together, these findings suggest that prolonged sensory and environmental deprivation acts as a chronic stressor capable of dysregulating the HPA axis and altering cortisol rhythmicity, whereas short-term or voluntary forms appear to have limited or adaptive effects. Duration and environmental context therefore emerge as critical determinants of neuroendocrine outcomes.

### 3.3. Neuroimmune System

Sensory deprivation can trigger significant neuroimmune changes in the brain. Male C57BL/6J mice subjected to 2–4 weeks of social isolation show an increase in cortisol levels, activation of the NF κB pathway and microglia, changes in brain morphology (density, branching, lacunarity) with reduced exploratory behaviors and increased anxiety levels [60]. One study compared two groups of mice, the first consisting of individuals raised in isolation and the second raised in pairs, both subjected to an 8 min ischemic stroke. The study showed that prior to ischemia, the socially isolated mice exhibited increased MHC-II expression in the cortical and hippocampal areas, suggestive of microglia sensitization. After 24 h from ischemia, these mice developed increased expression of IL-1β in the hippocampus and of TNF-α, IL-1β, and IL-6 in cortical regions, compared to pair-housed mice [61]. In a 2024 study, post-weaning isolation increased the levels of pro-inflammatory cytokines (e.g., IL-1α, IL-1β, IL-13, IL-17, TNF-α, IFN-γ), as well as activation of the nuclear factor kappa-light-chain-enhancer of activated B cells (NF κB) pathway [62]. In humans, some data are derived from space mission simulations carried out in healthy volunteers, and from astronauts after space missions [63,64,65]. After 9 days of confinement, a reduction in absolute and relative terms of both innate and adaptive immunity cells can already be observed [66]. After 17 days, there is a marked decrease in monocytes with Toll-like receptors (TLR3, TLR4, TLR5, TLR6, TLR8, TLR9) and an increase in plasmacytoid dendritic cells (CD14, CD16, CD123, CD85k), important in the production of interferons [67]. The most significant data come from the above-mentioned MARS 500 study [47,68,69,70]. The main findings were an increased production of cytokines in response to Epstein–Barr Virus (EBV) particles, as well as of IFNγ and TNF [70].

Antarctic expeditions represent a natural model of immune adaptation to ICE conditions [71]. Studies report stress-related immune alterations, including increased cortisol levels, NK cytotoxicity and T-cell activation during deployment, followed by post-expedition leukopenia and impaired leucopoiesis [71,72]. Seasonal suppression of mucosal IgA/IgM, reduced T-cell proliferation, cytokine dysregulation, and increased EBV reactivation were also documented [71,73,74,75]. While short-term missions mainly induce microbiome fluctuations and pro-inflammatory responses, repeated or prolonged exposures may exacerbate mitochondrial dysfunction in leukocytes, suggesting cumulative immune and metabolic stress [76,77]. An interesting study assessed immune functions in 14 members staying in Concordia station [78]. The results showed that in the first two months there was a moderate increase in the global immune functions, while after three–four months cytokine responses were markedly increased, with changes in gene transcription levels possibly driven by hypoxia [78].

Taken as a whole, these findings would indicate that Antarctic residence seems to induce a transient immune alteration with no sign of immunodeficiency, returning to normal levels when the crew members end the mission and return to non-isolated environments [79].

### 3.4. Circadian Rhythms: Impact of Sensory Deprivation

Sensory deprivation may significantly impair and/or disrupt circadian rhythms. The absence or reduction in external zeitgebers, such as light, sound, and social cues, impairs the entrainment of the suprachiasmatic nucleus (SCN), the brain’s master clock, leading to internal desynchronization across physiological systems [80,81,82]. This disruption modifies the regulation of circadian rhythms and produces a state of internal desynchronization across multiple physiological systems, including the sleep–wake cycle, melatonin and cortisol secretion, and body temperature regulation [83,84,85]. Experimental and clinical studies show that sensory-deprived individuals, such as totally blind people, isolated individuals (e.g., prisoners, astronauts, speleologists, soldiers in a bunker), and ICU patients, frequently develop sleep–wake disorders, flattened cortisol profiles, and reduced amplitude of circadian gene expression [86,87,88,89,90,91,92,93,94,95,96,97]. These circadian disruptions are supposed to be at the basis of the psychological and cognitive symptoms frequently observed in sensory deprivation. Dysregulated sleep–wake cycles and hormonal rhythms are associated with increased risk of depression (particularly in individuals who lack external feedback to anchor their sense of continuity and self), anxiety, and cognitive impairments [83,89,94,97]. Furthermore, the loss of temporal structure, a direct consequence of circadian misalignment, may contribute to the disturbance of time perception, a common feature in sensory deprivation that underlies phenomena such as derealization, disorientation, and a pervasive sense of suspension or unreality [98,99,100].

### 3.5. Structural Brain Changes

Brain plasticity allows the central nervous system (CNS) to change its structures and functions in response to a variety of both endogenous and exogenous stimuli, as well as damage or insults [101]. This sort of adaptation appears to be crucial in our species as learning and the cultural transmission of information require a versatile brain structure [102,103,104]. These abilities of the cerebral cortex are particularly emphasized when such stimuli are lacking. The available literature indicates that subjects deprived of congenital or acquired auditory or visual input show a functional reorganization of the cortical areas affecting both lost and intact sensory capacities [105,106,107,108,109,110,111,112]. The ensuing changes are called cross-modal plasticity [105,106,107]. This activity implies that the cortical areas devoted to the lost sense are being recruited by the sense that is still intact, e.g., hearing. According to the so-called *perceptual deficit hypothesis*, the deprivation of one sense results in deficits of other sensory modalities, while the *sensory compensation hypothesis* holds that the deficit of one sensory area leads to overcompensation by other senses [113,114,115]. In any case, both options are considered too simplistic [106,116,117].

Since Hubel & Wiesel’s pioneering studies, mammal models have been used to study brain plasticity following deprivation of a sense [118,119,120,121], and it was soon demonstrated that it was present in both young and adult individuals [122,123,124,125,126]. Cross-modal neuroplasticity, while most prominent in early-onset sensory deprivation, also occurs in individuals who experience sensory loss later in life, albeit with distinct patterns and limitations [127]. In late-onset blindness, the visual cortex can still undergo reorganization, although typically to a lesser extent than in congenitally blind individuals [128]. Studies using functional magnetic resonance imaging (fMRI) reported that the occipital cortex in late-blind individuals may become responsive to tactile and auditory stimuli, particularly when these inputs are behaviorally relevant, such as during Braille reading or sound localization [129,130,131]. The same finding was reported in normally sighted subjects blindfolded for 5 days [132]. In late-onset deafness, reorganization of the auditory cortex is similarly limited compared to early-onset cases, although compensatory changes can still occur, especially in response to visual cues and during speech-reading tasks [133]. In deaf adults with cochlear implantation, increased visual activation in auditory areas does not even hamper, but predicts better hearing outcomes post-implantation. Cross-modal plasticity may therefore be adaptive, not maladaptive [134]. These findings indicate that while the adult brain retains some capacity for cross-modal plasticity, its extent and functional significance depend on the age of deprivation onset, the individual’s sensory experience history, and task-specific demands [134].

However, exposure to environments lacking significant social and sensory input may induce significant detrimental effects on gray matter volume. Some evidence derives from studies conducted on members of Antarctic expedition crews, and in astronauts following space missions or living a long time in the International Space Station [135], with situations replicating being isolated, confined, and ICE. Prolonged Antarctic isolation has been associated with structural brain changes, particularly gray matter reduction in regions critical for cognitive processes [21,136,137,138]. A 14-month mission at Neumayer III, a station located on the Ekström Ice Shelf in the Atlantic sector, revealed a significant decrease in dentate gyrus volume (7.2 ± 3%), lower levels of brain-derived neurotrophic factor (BDNF), an important neurotrophin, and declines in spatial processing and selective attention [138]. Similarly, MRI studies at Concordia Station, located on a site called Dome C on the East Antarctic plateau at an altitude of 3230 m, crew members showed reductions in gray matter in the hippocampus, temporal and parietal lobes, and pallidum, with a partial recovery after five months from the end of the mission; sleep quality emerged as a protective factor against volume loss [137]. Not surprisingly, another recent study reported similar results in 17 astronauts (9 men and 8 women), during a six-month mission on the International Space Station (ISS). Participants, who underwent MRI scans before and after the mission, showed a significantly decreased volume of the entire left hippocampus, particularly of its anterior subregion, and of the body subregion of the right hippocampus [139]. Sex-specific differences were also observed: male astronauts experienced a much larger reduction in right hippocampal volume (about −2.247%) than females (about +0.271%), but not in the left hippocampus or its subregions [139]. However, importantly, it should be underlined that the observed alterations are probably the result of the combination with other stressors, such as microgravity, radiation exposure, isolation, circadian rhythm disruption, and elevated CO_2_ levels [140,141,142]. The findings of other studies reported upward brain shift, alterations in ventricular volumes and cerebrospinal fluid (CSF) distribution, central sulcus narrowing, and cerebral aqueduct twisting [140,141,143,144,145,146]. A marked reduction in gray matter (GM) was widely described, particularly in the frontal and temporal lobes, accompanied by localized bilateral increases in the medial primary somatosensory and motor cortices, possibly related to the novel sensory and motor demands due to microgravity [140,147,148]. Most GM changes reflect volumetric variations rather than actual tissue loss and appear to be partially or fully reversible after return to Earth [140,141,144]. Alterations in white matter (WM) have been widely reported, such as an increase in the inferior cerebellar peduncle, areas surrounding the precentral and postcentral gyri, superior/inferior longitudinal fasciculi, optic radiations, and a decrease in the temporal and occipital lobes. However, post-flight and follow-up (7 months after Earth return) comparisons revealed opposite findings. Notably, the cerebellar WM increase persists for up to seven months after returning to Earth [144]. The hippocampus of astronauts, similarly to that of people on Antarctic missions and animal models, may also be susceptible to change, including reduced production of BDNF, decreased neural plasticity and neurogenesis, and hippocampal atrophy [138,149,150,151,152,153,154,155]. Structural and/or functional alterations in the hippocampus may negatively affect memory, learning abilities, spatial orientation, and adaptability, potentially leading to difficulties in executing complex tasks [140,141]. Functional brain changes have also been reported mainly following spaceflights, in networks involving motor, somatosensory, cerebellar, vestibular, and visual regions, like insular cortex and supramarginal gyrus, critical areas for multisensory integration and motor control [140,156,157]. In astronauts, the functional brain connectivity seems to adapt to microgravity-induced sensory deprivation, facilitating adjustment to altered sensory contexts, but simultaneously contributing to sensorimotor or perceptual difficulties upon return to Earth [140].

Taken together, the findings of these studies in humans, paralleling those observed in animal models, suggest that environmental monotony, confinement, and limited social interaction can compromise hippocampal plasticity and brain, potentially impairing cognition, especially during long-term missions [21,158,159,160].

## 4. Psychological and Psychopathological Effects of Sensory Deprivation

The literature indicates that sensory deprivation may significantly impair psychological wellbeing and contribute to the onset or exacerbation of psychopathological disorders, including depression, anxiety, hallucinations, symptoms of derealization and/or depersonalization, as well as cognitive deficits [4,161,162,163,164,165].

The association between sensory deprivation and depression is well established and extensively documented [4,166,167,168,169,170,171]. Several longitudinal and cross-sectional studies confirm that single or dual sensory loss significantly increases the risk of depression [161,166,167,168,170,172,173,174,175,176]. Although hearing and vision loss are the most extensively studied, some research indicated that loss of smell (anosmia) and/or taste is also linked to increased loneliness, reduced overall wellbeing, and heightened depressive symptoms [4,177,178,179,180,181,182]. The connection between sensory deprivation and depression is further supported by studies showing that even a few days or weeks of reduced environmental and/or social stimulation can lead to the emergence of depressive symptoms and demotivation [162]. This phenomenon is particularly evident in specific populations such as incarcerated individuals, hospitalized patients, polar expedition researchers, and individuals experiencing social isolation [21,162,183,184,185,186,187]. In polar explorers a specific cluster of affective symptoms have been reported called Polar T3 syndrome or winter-over syndrome or subsyndromal seasonal affective disorder, characterized by lowered mood, irritability, apathy, anhedonia, social withdrawal and cognitive decline particularly among individuals with pre-existing vulnerabilities or psychological predispositions [164,188]. Similar symptoms have also been observed in astronauts, together with concentrating impairments and more marked sleep disturbances [189,190,191].

Several mechanisms may link sensory loss to mood disorders: (1) social isolation (sensory deficits reduce social participation, limit interpersonal interactions, and increase loneliness and feelings of exclusion) [169,192]; (2) limitations in daily activities (restrictions resulting from sensory impairments negatively affect autonomy, self-efficacy, and self-esteem, which in turn contribute to depressive symptoms) [161,167,173,174,193,194,195]; (3) psychosocial factors (these include the loss of meaningful roles, communication difficulties, reduced engagement in rewarding activities, and a diminished sense of purpose) [168,196]; (4) bidirectional relationships (sensory loss increases vulnerability to depression, while psychological distress worsens the perception of and adjustment to sensory impairments) [167,170,193].

Anxiety is also strongly associated with sensory loss or sensory deprivation [175]. Both sensory deficits and reduced environmental stimulation are linked to a heightened risk of anxiety, especially in cases of dual sensory loss (DSL) [3,161,166,168,173,175,176,197,198]. Experimental studies inducing sensory deprivation (e.g., reduced environmental stimulation, temporary auditory/visual isolation) documented the emergence of anxiety symptoms such as agitation, anticipatory anxiety, panic-like experiences, or marked distress, particularly in individuals with high trait anxiety or fantasy proneness [162,165,199]. Furthermore, sudden sensory loss (e.g., due to accidents or trauma) or extended exposure to monotonous and socially deprived environments (e.g., prolonged hospital stays, COVID-19-related isolation, incarceration, polar expeditions) can lead to generalized anxiety, social phobia, and panic attacks [21,162,184,185,186,187,200].

Sensory deprivation or loss is also strongly associated with an increased risk of perceptual disturbances, such as hallucinations, derealization, and cognitive deficits [162,171,201]. According to the *deafferentation theory*, when a sensory input is reduced or absent, the corresponding cortical areas (often referred to as “orphaned” due to conditions such as deafness or blindness) become hyperexcitable and may spontaneously generate perceptions in the absence of external stimuli [202,203]. Among patients with visual impairments, albeit with no cognitive deficits or psychiatric disorders, a common phenomenon called Charles Bonnet Syndrome (CBS) or “phantom vision” may develop, consisting of complex visual hallucinations [4,202,204,205,206]. Similarly, individuals with hearing impairments often report hallucinations known as “musical ears syndrome” involving sounds such as voices, noises, or music, occurring in the absence of delusions or formal thought disorder, positively related to the severity of the sensory loss [202,204,205,207,208]. Sensory and ideational hallucinations are frequently reported as consequences of prolonged isolation such as incarceration or involuntary hospitalization. The hallucinations of socially deprived prisoners may be vivid and are referred to as “prisoner’s cinema”. Generally, they are fearful and distressing, and trigger traumatic consequences, while rarely are they comforting [162,163,200,209,210]. It should also be highlighted that sensory deprivation experiences can be pursued for religious scopes so that hallucinations are interpreted within the spiritual domain.

Acute sensory deprivation experiments (e.g., anechoic chambers, temporary auditory/visual isolation) in healthy individuals rapidly induced visual, auditory, or tactile hallucinations, as well as distortions in bodily perception and time, including out-of-body experiences [18,162,164,165,211,212,213].

The explanatory hypothesis of these phenomena is that during sensory deprivation, the brain, albeit with no stimuli reaching it, continues to process information and create hallucinations for its basic need to provide explanations and meaning, a function called *apophenia* that is the attribution of meaning and links between unrelated objects [214].

Reduced environmental stimulation also increases the likelihood of developing dissociative symptoms, such as derealization (a sense of detachment from the external world) and depersonalization (a sense of detachment from the self). Studies on individuals with auditory, visual, vestibular, or other sensory dysfunctions report more frequent and severe derealization and depersonalization symptoms compared to individuals without such impairments [4,215,216,217]. This can be due to the discrepancy between expected and actual sensory input, which disrupts multisensory integration. Moreover, individuals exposed to sensory and environmental isolation (e.g., sensory-minimized rooms, incarceration, polar expeditions, prolonged hospitalization) frequently report sensations of unreality, suspension, and alienation from their bodies and the external world [162].

Reduced sensory stimulation is associated with a rapid decline in cognitive functioning, probably due to a decline in neural plasticity and activity [171,218]. Sensory deprivation has been broadly associated with impairments in attention and memory, executive dysfunction, difficulties in concentration, and disturbances in abstract thinking, with risk and severity of cognitive decline correlating with the duration and intensity of sensory deprivation [171,218,219,220]. Several longitudinal studies suggest that reduced environmental/social stimulation and/or age-related hearing, visual, or olfactory loss constitute significant risk factors for cognitive decline and the development of dementia [220,221,222,223,224]. Individuals with hearing loss exhibit a 24% higher risk (HR = 1.24) of cognitive impairment compared to those with normal hearing [225]. Furthermore, dopaminergic dysfunction-related conditions (such as Parkinson’s disease, schizophrenia, ADHD, and autism) are often characterized by significant olfactory impairments [4,226,227]. Incidentally, the frequent loss or impairment of olfaction after COVID-19 pandemic is an open issue that deserves to be carefully addressed in light of our previous considerations [228,229,230,231].

Available studies involving isolated prisoners, polar explorers, individuals in states of prolonged immobility or sensory monotony showed declines in cognitive performance, distortions perception, and increased vulnerability to cognitive errors [162,232].

Individual differences seem important to mediate the impact of sensory deprivation. They include traits such as conscientiousness, openness to experience, extraversion, emotional stability, strong coping and adaptive capacities, resilience, and preserved cognitive functioning, which are associated with both a lower prevalence of sensory impairment and a reduced negative psychological impact [162,233,234]. Conversely, neuroticism, psychological fragility, rigidity, and passivity are traits associated with worse psychological outcomes following sensory loss [233,234,235]. In addition, individuals who perceive sensory deprivation as a threat are more likely to experience negative psychological reactions, whereas those who interpret it as a neutral, temporary, and/or necessary condition (e.g., in the context of illness) tend to report more tolerable or even positive experiences, even when hallucinations or perceptual disturbances may occur [162]. Nevertheless, prolonged isolation remains detrimental to mental wellbeing, even in individuals who are psychologically prepared for or predisposed to tolerating such conditions. Not surprisingly, individuals with pre-existing psychiatric disorders or traits (such as borderline, obsessive, or psychopathic personality features) are at greater risk of developing severe psychopathological symptoms, including hallucinations, paranoia, and loss of contact with reality during episodes of sensory isolation [163,235]. Individuals with high levels of schizotypy or a predisposition to fantasy proneness and increased suggestibility also show greater susceptibility to developing hallucinations and unusual perceptual experiences under conditions of experimental sensory deprivation [1,2,18,236].

## 5. Discussion and Conclusions

Adaptation to changing internal and external environmental conditions has permitted the survival of humans throughout harsh periods, such as the last glacial maximum, or extreme heat. Coping with adverse and even extreme conditions has led to the onset of novel strategies, behaviors and tools to face environmental challenges. Therefore, the constant interaction with the physical and social environment is at the basis of our evolution, given the flexibility of our brain derived from its functional and structural plasticity [237,238,239,240]. A balanced environment is one that ensures a continuous flux of sensorial stimuli and relationships with others. When the stimuli decline or vanish, as it may happen in conditions of sensory deprivation, the ability of our brain to adjust to novelties is impaired or lost [116,241,242,243,244]. Variables such as duration, individual predisposition, and willingness to do the experience are likely to shape the outcomes [245,246,247]. It is obvious that the effects of a voluntary sensory deprivation, such as that of hermits or members of religious sects seeking and/or practicing a lonely life, may be totally different from that of patients in IUs and isolated hospital wards, or from that deliberately used to interrogate prisoners or as a form of torture [13,17,248].

The available literature suggests that duration of sensory deprivation is a critical factor to determine its positive or negative effects. Indeed, short-term, voluntary experiences in float tanks may be beneficial to restore psychological wellbeing, to induce relaxation and reduce pain. By contrast, nowadays international and national laws have banned the use of sensory deprivation to interrogate or punish prisoners, given its long-term negative psychopathological consequences including not only anxiety and depression but also more severe symptoms such as derealization, hallucinations, speech difficulties and personality changes [4,18,164,249,250,251]. Indeed, prolonged sensory deprivation in interrogation and detention settings has been widely classified as a form of psychological torture and is prohibited under international human rights law, including the United Nations Convention Against Torture (UNCAT).

The current and renewed interest in sensory deprivation derive from data gathered from individuals facing extreme environmental conditions, typical of spaceflights, polar expeditions, submarines, sea surviving, cave explorations, long-term working in front of a radar or a screen, or clinical contexts, such as that of ICU or isolated wards (as those of the COVID-19 time) [3,5]. Although data are scattered and gathered in small samples, they suggest that sensory deprivation may impair stress processes and neuroplasticity, as well elicit a state of chronic neuro-inflammation that would underline the different psychopathological and cognitive symptoms reported [6,7,8]. All together, these findings and the current impulse to future deep-space exploration and the increasing number of missions in extreme environmental conditions require a deep and perhaps mandatory understanding of the neural vulnerabilities that, in our opinion, are critical to developing countermeasures to prevent and/or protect the health of astronauts and explorers. The next space missions include the Artemis program (the return to the moon that will for the first time include a woman), SpaceX’s starship, NASA’s aim to carry humans to Mars, the Europa Clipper, ExoMars working on new rockets, robotic planetary science and some others in the next decade. The missions in Antarctica investigate adaptation to extreme cold and isolation, climate change and astrophysics parameters. Interestingly, scientists in Concordia and McMurdo stations are simulating Mars conditions to explore the consequences of long-term spaceflights.

In both these two extreme conditions, preventive measures should include environmental enrichment with natural sounds (birds, sea waves, cascades, rains) for their stress-reducing effects [252], maintenance of circadian rhythms, good sleep, constant eating times [253,254,255,256,257], connections with loved ones, physical activity, and especially good training to increase resilience and coping strategies. If necessary, online supporting psychotherapy should be provided.

Across the heterogeneous models reviewed, several converging findings emerge: prolonged sensory and environmental under-stimulation consistently act as a chronic stressor associated with HPA axis dysregulation, neuroimmune activation, circadian desynchronization, hippocampal vulnerability, cognitive decline, and increased risk for affective and perceptual disturbances. By contrast, short-term and voluntary sensory restrictions appear to engage adaptive regulatory mechanisms and may promote relaxation and emotional modulation. However, important methodological gaps persist. Direct human neurobiological studies under controlled multisensory deprivation are scarce due to ethical and technical constraints; most evidence derives from proxy models (social isolation, unimodal loss, or extreme environments with multiple confounders). There is also a lack of standardized duration thresholds, limited longitudinal data, and insufficient integration between neurobiological and clinical outcomes.

Future research should prioritize controlled translational paradigms, multimodal neuroimaging, biomarker-based studies, and the identification of individual vulnerability and resilience factors, particularly in contexts of prolonged environmental deprivation such as spaceflight and polar missions.

In conclusion, the present review, rather than providing a comprehensive mechanistic model, aimed to map the empirical landscape of research on sensory under-stimulation and its psychological consequences, to identify converging findings, methodological gaps, and areas that warrant further studies, as well as to suggest some preventive measures at times of these novel space and Antarctica missions.

## Data Availability

No new data were created or analyzed in this study.
